# Recurrence of paraproteinemic crystalline keratopathy after corneal transplantation: A case of monoclonal gammopathy of ocular significance

**DOI:** 10.1016/j.ajoc.2020.100803

**Published:** 2020-07-02

**Authors:** Siamak Nobacht, Benno Kusters, Myrte B. Breukink, Gerard A. Rongen, Johannes R.M. Cruysberg

**Affiliations:** a Department of Ophthalmology, Radboud University Medical Center, Nijmegen, the Netherlands; b Department of Pathology, Radboud University Medical Center, Nijmegen, the Netherlands; c Department of Internal Medicine, Radboud University Medical Center, Nijmegen, the Netherlands

**Keywords:** Crystalline keratopathy, Paraproteinemia, Monoclonal gammopathy, Undetermined significance, Ocular significance

## Abstract

**Purpose:**

To report the long-term follow-up (12 years) of a 36-year-old male patient with crystalline keratopathy of both eyes, diagnosed with monoclonal gammopathy of undetermined significance (MGUS). Complete ophthalmic, systemic, and corneal immunohistochemical evaluations were performed.

**Observations:**

Slit-lamp examination revealed bilateral fine iridescent confluent crystalline deposits in all layers of the cornea, both peripherally and centrally. Systemic evaluation revealed abnormal M protein, IgG-kappa type, in blood and urine. Bone marrow aspiration showed a monoclonal plasma cell concentration of 2%. Consequently, the patient was diagnosed with MGUS. Because of progressive bilateral visual loss in the following 10 years, a perforating keratoplasty was performed on the left eye. Immunohistochemical analysis of the native cornea (the corneal button) revealed depositions of the same M protein type as detected in plasma and urine. Electron microscopy showed rhomboid-shaped corneal deposits of various sizes up to 4 μm. Recurrence of crystalline keratopathy was observed 9 months after keratoplasty. The monoclonal protein remained stable and the MGUS did not progress to multiple myeloma nor a related disorder.

**Conclusions and importance:**

Crystalline keratopathy may be associated with MGUS in otherwise healthy individuals. If the keratopathy causes binocular visual loss, a corneal transplantation may be required. Unfortunately, recurrence of crystalline deposits in the corneal graft may occur within one year. This suggests that patients with vision impairment due to paraproteinemic keratopathy who are diagnosed as MGUS, in fact, have a monoclonal gammopathy of ocular significance (MGOS).

## 1 Introduction

Monoclonal gammopathy of undetermined significance (MGUS) is a proliferative plasma cell disorder, characterized by the presence of a monoclonal (M) protein spike of ≤30 g/l, a plasma cell content of <10% in bone marrow, and the absence of multiple myeloma or related lymphoplasmatic malignancies (LPMs).^1^ The updated disease definition of multiple myeloma includes validated biomarkers in addition to existing CRAB features (hyperCalcemia, Renal failure, Anemia and Bone lesions).^1^ Regular clinical evaluations are necessary to monitor the disease state. If there is no progression to multiple myeloma or serious B-cell disorders, MGUS does not require treatment. Patients with MGUS have been reported to develop paraproteinemic keratopathy, which may require keratoplasty when significant visual loss occurs.^2^, ^3^, ^4^, ^5^ The aim of this study is to describe the clinical characteristics of a 36-year-old male patient with crystalline keratopathy associated with MGUS, with long-term follow-up (>12 years) of ocular and systemic features.

## 2 Case report

In 2006, a 36-year-old male patient was referred to the Department of Ophthalmology at the Radboud University Medical Center because of decreasing visual acuity of both eyes, associated with a corneal haze of unknown etiology. He had a history of recurrent anterior uveitis of the right eye, which was controlled with topical steroids. He was otherwise healthy and did not take any systemic medication. His family history was negative for corneal diseases.

**Fig. 1:**
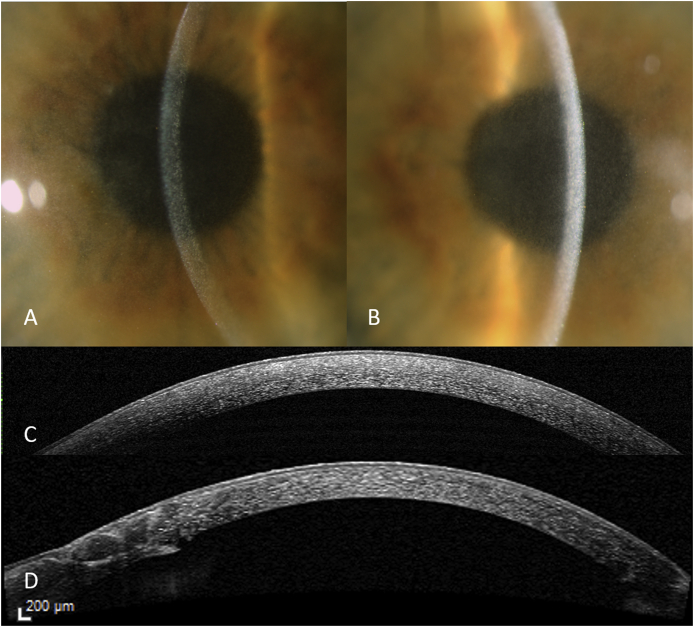
Slit-lamp photograph of the right eye (A), and left eye (B), showing corneal deposits in the whole cornea of both eyes. Optical coherence tomography of the left cornea, prior to corneal transplantation (C), and 9 months after corneal transplantation (D), showing recurrence of corneal deposits after transplantation.

**Fig. 2:**
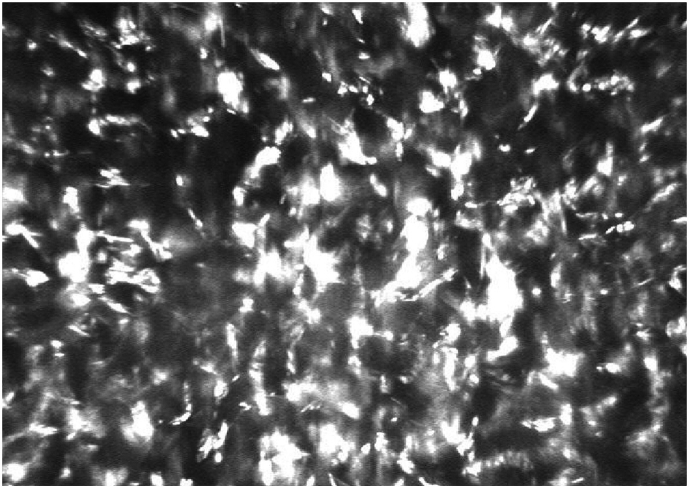
In vivo confocal microscopy of the RE, showing numerous highly reflective deposits in the corneal stroma.

The visual acuity of his right eye (RE) was 0.6 (6/10 Snellen) and of his left eye (LE) 0.8 (6/7.5 Snellen) without correction. The visual acuity did not improve with glasses. Slit-lamp examination revealed fine iridescent confluent corneal crystals in both eyes, distributed throughout all corneal layers, both peripherally and centrally (Fig. 1A, Fig. 1B). Mild anterior uveitis was observed in the right eye (Tyndall 1+; cells 1+). His lenses were clear and no abnormal ophthalmoscopic findings were observed. The intraocular pressures were normal, at 11 mm Hg in the RE and 10 mm Hg in the LE. Optical coherence tomography (OCT) revealed fine, hyper-reflective dot-shaped deposits across all layers of the cornea in both eyes (Fig. 1C). In vivo confocal microscopy (IVCM) showed highly reflective deposits throughout the cornea in both eyes (Fig. 2).

The patient was referred to the Department of Internal Medicine for systemic evaluations to exclude metabolic and hematologic disorders. Concentrations of cystine, oligosaccharides and mucopolysaccharides were in the normal range. The concentration of cystine in granulocytes was 0.01 mmol/mg (normal <0.43 mmol/mg). Serum electrophoresis with immunofixation showed an IgG kappa M protein at a concentration of 3.9 g/l. Renal functions were normal. Bone marrow aspiration showed an increased monoclonal plasma cell concentration of 2% (<10% in MGUS). The systemic findings established the diagnosis of a monoclonal gammopathy of undetermined significance (MGUS), IgG kappa type, for which no systemic treatment is required. Based on the ocular characteristics, a diagnosis of crystalline keratopathy associated with MGUS was established. The patient returned to his referring ophthalmologist for further observation.

Ten years later, in 2016, the (46-year-old) patient was referred back to our clinic because of progression of his visual loss. His best-corrected visual acuity was 0.4 (6/15 Snellen) with S+1.00 = C-1.00 axis 10° in the RE and 0.3 (6/20 Snellen) with S-2.00 = C-1.50 axis 100° in the LE. The corneal deposits had increased considerably in both eyes. Follow-up of the systemic findings during the past 10 years showed stable circulating M protein levels (range, 3.1–3.9 g/l), which is within the range of MGUS (≤30 g/l). Renal functions remained normal. A corneal transplantation (full thickness keratoplasty) was performed on the LE.

**Fig. 3:**
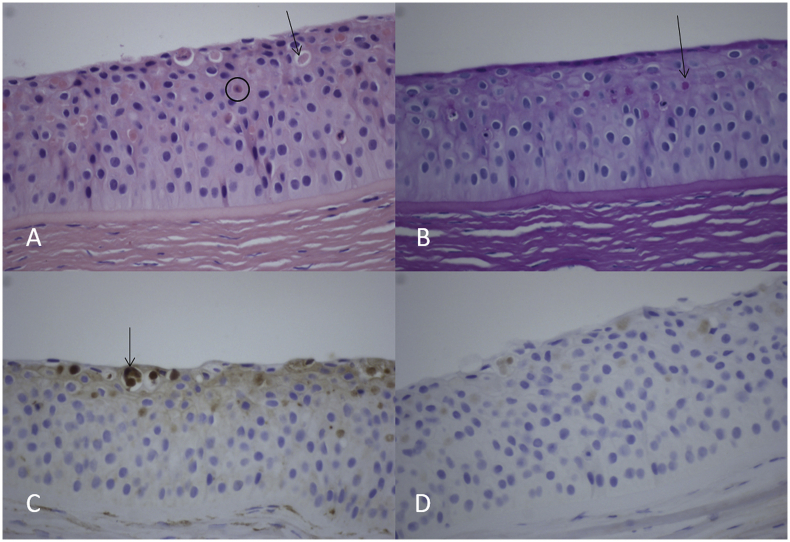
Histology of the left corneal button, showing eosinophilic intraepithelial inclusions that were visible among hematoxylin and eosin staining (A), and PAS staining (B). Examples are indicated by arrows. These globular inclusions stained positive in the kappa light chain staining (C), indicating a kappa restriction. Lambda light chain staining exhibited only a faint back-ground staining (D). In hematoxylin and eosin staining (A), occasional apoptotic keratinocytes were present (black encircled cell).

**Fig. 4:**
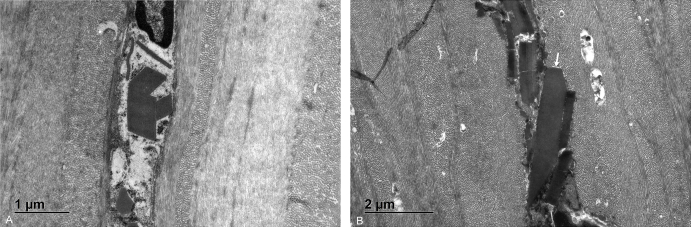
Corneal crystalline deposits on electron microscopy, showing multiple rhomboid-shaped and rod-shaped inclusions (A). The size varied between 500 nm up to 4 μm (B).

Histological analysis of the corneal button exhibited eosinophilic intraepithelial inclusions that were visible among hematoxylin and eosin (HE) staining and Periodic acid-Schiff (PAS) staining (Fig. 3A and B). These globular inclusions stained positive in the kappa light chain staining, indicating a kappa restriction (Fig. 3C). Control staining for lambda light chain exhibited only a faint background staining (Fig. 3D). In HE staining, occasional apoptotic keratinocytes were present (Fig. 3A). On electron microscopy, rhomboid-shaped inclusions of various sizes up to 4 μm were visible (Fig. 4). These histological analyses confirmed the initial diagnosis of crystalline keratopathy associated with MGUS.

After corneal transplantation surgery of the LE best-corrected visual acuity had improved to 0.7 (range, 6/10 to 6/7.5 Snellen). However, nine months postoperatively recurrence of corneal deposits appeared in the graft (Fig. 1D), with unchanged visual acuity at that time. Two years after corneal surgery, the corneal deposits had further increased in both eyes, associated with a declined best-corrected visual acuity of 0.3 (6/20 Snellen) in the RE and 0.5 (6/12 Snellen) in the LE.

## 3 Discussion

Monoclonal gammopathy-associated crystalline keratopathy was first reported by Meesmann in 1934 in a multiple myeloma patient.^6^ Extracellular deposition of M protein leads to crystal formation in the cornea. In the patient described here, two observations support a causal role of circulating M protein in the development of the observed keratopathy: 1) immunohistochemical analysis revealed that the circulating M protein was also present in the corneal depositions; and 2) after corneal transplantation, corneal depositions re-occurred and the circulating M protein concentration remained unchanged. In analogy to Koch's postulation, a definite proof of causality would be provided if reduction of circulating M protein would prevent re-occurrence after a corneal transplantation. It is unknown why patients with MGUS develop corneal deposits. It has been postulated that these crystalline deposits may be delivered from limbal vessels to the cornea.^3^ Theoretically, variation in biochemical properties of the M protein and/or differences in limbal vascular properties that contribute to the transport of circulating M protein to the cornea may explain the variability in corneal deposit formation among MGUS patients.^2^^,^^3^^,^^7^ Although endothelial dysfunction may also contribute to the development of corneal opacity, the association between corneal endothelial dysfunction and crystal deposition is not clear.^4^ A better understanding of the pathophysiological factors involved in corneal deposition of M protein could provide therapeutic options rather than corneal transplantation in these patients.

The incidence of crystalline keratopathy in patients with MGUS is not known. Bourne et al. reported an incidence of 1% among 100 patients with amyloidosis, of whom 23 were diagnosed as MGUS.^8^ Primary symptoms include corneal crystal deposits and corneal opacity. Corneal crystalline deposits may be the first clinical symptom of monoclonal gammopathy, and all layers of the cornea may be affected.^2^^,^^4^^,^^9^

The clinical manifestation of corneal deposits associated with MGUS may have a varied, chameleon-like appearance, as described by Lisch and coworkers.^10^ The classical manifestation is characterized by grey-white, grey-brown polychromatic and iridescent dot-like crystals in any layer of the cornea.^2^^,^^5^^,^^7^^,^^10^ In our opinion, when comparing the corneal crystals in cystinosis to those seen in MGUS-associated keratopathy, the corneal deposits in MGUS are more confluent, less iridescent and more visually significant.

In the case of bilateral corneal deposits in an otherwise young healthy individual, a hematological work-up is indicated to determine circulating M protein, and if present, to exclude a malignant plasma clone indicative of multiple myeloma. MGUS is a benign form of paraproteinemia. However, 10–18% of MGUS patients can develop multiple myeloma, macroglobulinemia, amyloidosis or lymphoma over the years.^9^ A systemic therapy is not indicated in MGUS, but annual hematological and blood analysis is advisable.

Unfortunately, there is no medical treatment available for a MGUS-associated crystalline keratopathy, neither locally nor systemically. Unlike monoclonal gammopathy of *renal* significance (MGRS), in which systemic therapy may be indicated, there is no evidence in MGUS patients that clone directed therapy can reduce the density of the deposits in the cornea or prevent the recurrence after corneal transplantation.^2^, ^3^, ^4^^,^^11^ Due to the significant impact of corneal disease on the quality of life in these patients, we suggest to introduce the diagnosis of monoclonal gammopathy of *ocular* significance (MGOS), on which future local or systemic therapy can be focused.

## 4 Conclusions

Crystalline keratopathy associated with MGUS is a serious ophthalmological complication and justifies the introduction of the term monoclonal gammopathy of *ocular* significance (MGOS) when it causes bilateral vision impairment. Unfortunately, recurrence of corneal crystalline deposits in the corneal graft may occur within one year and can currently not be prevented. Future therapeutic options to eradicate the plasma clone responsible for the circulating M protein could potentially reduce the risk of recurrence after full-thickness corneal transplantation. However, the complications associated with currently available systemic therapeutic options do not outweigh their potential benefit in patients with circulating M protein who develop corneal depositions.

## Patient consent

The patient provided written consent for publication of his case report in AJO Case Reports.

## Funding

No funding or grant support.

## Authorship

All authors attest that they meet the current ICMJE criteria for Authorship.

## Declaration of competing interest

The following authors have no financial disclosures: SN, BK, MB, GR, JC.
